# Generation of Individualized, Standardized, and Electrically Synchronized Human Midbrain Organoids

**DOI:** 10.3390/cells14151211

**Published:** 2025-08-06

**Authors:** Sanae El Harane, Bahareh Nazari, Nadia El Harane, Manon Locatelli, Bochra Zidi, Stéphane Durual, Abderrahim Karmime, Florence Ravier, Adrien Roux, Luc Stoppini, Olivier Preynat-Seauve, Karl-Heinz Krause

**Affiliations:** 1 Department of Pathology and Immunology, Faculty of Medicine, University of Geneva, 1206 Geneva, Switzerland; 2 Department of Medicine, Faculty of Medicine, University of Geneva, 1206 Geneva, Switzerland; 3 Laboratory of Biomaterials, Faculty of Dental Medicine, University of Geneva, 1206 Geneva, Switzerland; stephane.durual@unige.ch; 4 Laboratory of Toxicology and Therapeutic Drug Monitoring, Geneva University Hospitals, 1205 Geneva, Switzerland; 5 Tissue Engineering Laboratory, HEPIA HES-SO University of Applied Sciences and Arts Western Switzerland, 1202 Geneva, Switzerland; adrien.roux@hesge.ch (A.R.); luc.stoppini@hesge.ch (L.S.); 6 Department of Diagnostics and Department of Medicine, Geneva University Hospitals, 1206 Geneva, Switzerland

**Keywords:** pluripotent stem cells, organoids, neurospheres, cell therapy, Parkinson’s disease, air–liquid interface, 3D cell culture, spheroids, electrical recordings, AirLiwell

## Abstract

Organoids allow to model healthy and diseased human tissues. and have applications in developmental biology, drug discovery, and cell therapy. Traditionally cultured in immersion/suspension, organoids face issues like lack of standardization, fusion, hypoxia-induced necrosis, continuous agitation, and high media volume requirements. To address these issues, we developed an air–liquid interface (ALi) technology for culturing organoids, termed AirLiwell. It uses non-adhesive microwells for generating and maintaining individualized organoids on an air–liquid interface. This method ensures high standardization, prevents organoid fusion, eliminates the need for agitation, simplifies media changes, reduces media volume, and is compatible with Good Manufacturing Practices. We compared the ALi method to standard immersion culture for midbrain organoids, detailing the process from human pluripotent stem cell (hPSC) culture to organoid maturation and analysis. Air–liquid interface organoids (3D-ALi) showed optimized size and shape standardization. RNA sequencing and immunostaining confirmed neural/dopaminergic specification. Single-cell RNA sequencing revealed that immersion organoids (3D-i) contained 16% fibroblast-like, 23% myeloid-like, and 61% neural cells (49% neurons), whereas 3D-ALi organoids comprised 99% neural cells (86% neurons). Functionally, 3D-ALi organoids showed a striking electrophysiological synchronization, unlike the heterogeneous activity of 3D-i organoids. This standardized organoid platform improves reproducibility and scalability, demonstrated here with midbrain organoids. The use of midbrain organoids is particularly relevant for neuroscience and neurodegenerative diseases, such as Parkinson’s disease, due to their high incidence, opening new perspectives in disease modeling and cell therapy. In addition to hPSC-derived organoids, the method’s versatility extends to cancer organoids and 3D cultures from primary human cells.

## 1. Introduction

Organoids offer a ground-breaking opportunity to model healthy and diseased human tissue. They have attracted considerable attention because of their ability to mimic the natural cell-to-cell and cell-to-matrix interactions, and to represent cell functions closer to the in vivo situation. At least to some extent, organoids have the potential to replace two-dimensional (2D) cell cultures as well as animal experimentation. Today, organoids play a crucial role in advancing medical and life science research areas including basic research, functional genetics, toxicology, pathophysiology, and drug screening. Organoids also have a major potential as starting material for cell therapies.

Midbrain organoids are highly relevant in neuroscience and neurodegenerative disease research due to their potential for modeling complex brain functions and diseases. They are enriched in dopaminergic neurons. In the intact brain, these dopaminergic neurons will extend their axons into the striatum, where they release dopamine and thereby contribute to the fine-tuning of motor functions. Midbrain organoids provide a three-dimensional model for several in vitro studies [1], since they mimic the human midbrain at cellular, structural, and functional level [1,2,3]. These miniature structures allow to investigate neural development, diseases like Parkinson’s, bridging the gap between animal experiments and human trials [4]. They also allow to test the efficacy and/or potential neurotoxic effects of new drugs [5,6]. Midbrain organoids also allowed to generate patient-specific organoids to understand disease progression and investigate potential therapeutic strategies [7] as well as to develop cell therapies [8]. They can be exposed to various environmental toxins or stress to study their potential neurotoxic effects, aiding in understanding environmental contributions to conditions like Parkinson’s. Another promising application of midbrain organoids is the production of dopaminergic neurons for cell replacement therapy (e.g., Parkinson’s disease) [9,10,11].

Several methods showed the possibility to engineer organoids, including the hanging drop method [12] self or forced aggregation methods [13,14], micro modeling microwell plates [15,16,17,18], magnetic levitation [19], or encapsulation in scaffolds [20,21,22,23,24,25,26].

One of the standard methods to generate and cultivate midbrain organoids is an immersion/suspension cell culture technique previously described [27,28]. This method has several limitations, including the need for continuous agitation [28,29], lack of standardization mainly due to fusion between organoids [29,30,31,32], as well as the need for a high volume of culture media [28]. The continuous agitation submits cells to shear stress that may influence cell physiology [29,33]. Moreover, organoids in this method and all conventional methods in immersion often suffer hypoxia due to limited oxygen access [29]. Previously, 3D models including organoids have faced limitations in providing adequate nutrient and oxygen access [34,35]. This hypoxia leads to significant metabolic shifts, affecting various cellular pathways, differentiation, and proliferation [34,35,36,37]. Brain organoids are particularly susceptible to these nutrient deficiencies due to their size and complexity [38]. Recent studies in brain organoids have demonstrated that restoring oxygen levels improved neuronal survival and axon outgrowth [29,39,40,41]. These studies open new avenues for brain modeling [39,40]; however, “robust, reproducible, and scalable” methods of production according to industrial and clinical standards are still lacking [42].

This study aims to optimize a robust and reproducible method for generating standardized midbrain organoids, which can serve as tools for future applications in disease modeling, drug screening, and cell therapy.

## 2. Materials and Methods

### 2.1. Generation and Culture of Human Brain Organoids

#### 2.1.1. Embryonic Stem Cell Culture and Differentiation

The human embryonic stem (hESCs) cell line HS420 (Gift from Dr Outi Hovatta, Karolinska institute, Sweden) was cultured in Stemflex medium (Thermofisher, Geneva, Switzerland) on laminin 521-coated tissue culture flasks (Thermofisher) according to manufacturer’s instructions. HS420 cells at 70% of confluency were enzymatically passaged using Accutase (Thermofisher, Geneva, Switzerland, ref 00-4555-56) in 3D cell culture plates in X-VIVO medium (LONZA, Bâle, Switzerland, ref BE04-380Q) supplemented with 1% of PenStep, ROCK inhibitor (Y27632, aback) at 10 μM, LDN193189 at 0.5 μM and SB431542 at 10 μM.

#### 2.1.2. Midbrain Organoids Culture and Differentiation

**Figure 1:**
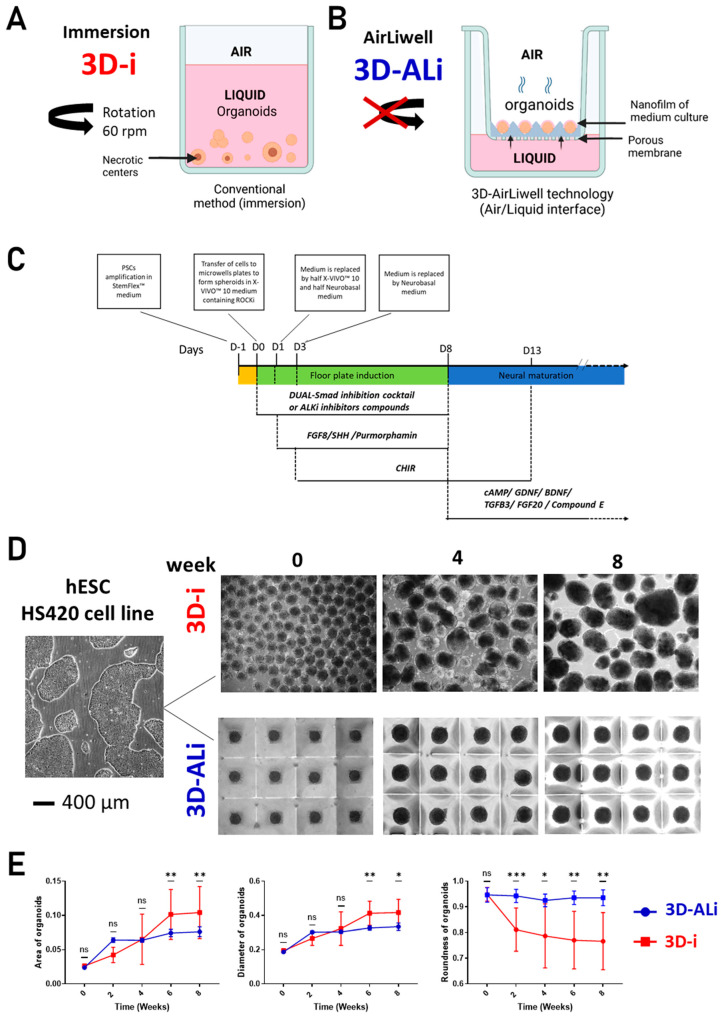
Highly standardized midbrain organoids generated at air–liquid interface: Illustration of the two 3D cell culture methods: (**A**) immersion method, (**B**) AirLiwell method. (**C**) Illustration of the protocol used to differentiate human pluripotent stem cells (ePSC) into midbrain neurons. Effect of the time culture of dopaminergic differentiation on organoid morphology. (**D**) Light microscopic photos showing the evolution of organoids during two months (60 days of differentiation) in 3D-ALi compared to 3D-i. The scale bar (left image) is valid for all pictures in panel D and corresponds to 400 μm. (**E**) Graphs representing the area, roundness, perimeter, circularity, and diameter of midbrain organoids (derived from hPSC, n = 12 organoids quantified for each time point) cultivated in 3D-i and 3D-ALi methods, with statistical significance (*p*-Value = *p*) indicated as follows: ns *p* > 0.05 non-significant, * *p* < 0.05, ** *p* < 0.01, *** *p* < 0.001.

Midbrain organoids were generated by using the following two different 3D cell culture techniques. The first one was the standard method “immersion” (3D-i); the organoids were first force-aggregated in plastic microwells, then removed and transferred in standard culture plates maintained under agitation (Figure 1A). In the second technique used, called the “AirLiwell” method, the organoids were forced to aggregate in non-adhesive microwells molded in medium-permeable agarose. In contrast to immersion, they were individually maintained into the microwells without any transfer, immersion, and agitation. A hemi-permeable membrane at the bottom of the molded microwells allows for their long-term stability and to establish air/liquid interface conditions that favor gas exchanges with air (Figure 1B).

##### Generation and Culture of Midbrain Organoids by Forced Aggregation Followed by Immersion (3D-i) [27,28]

**Scheme 1:**
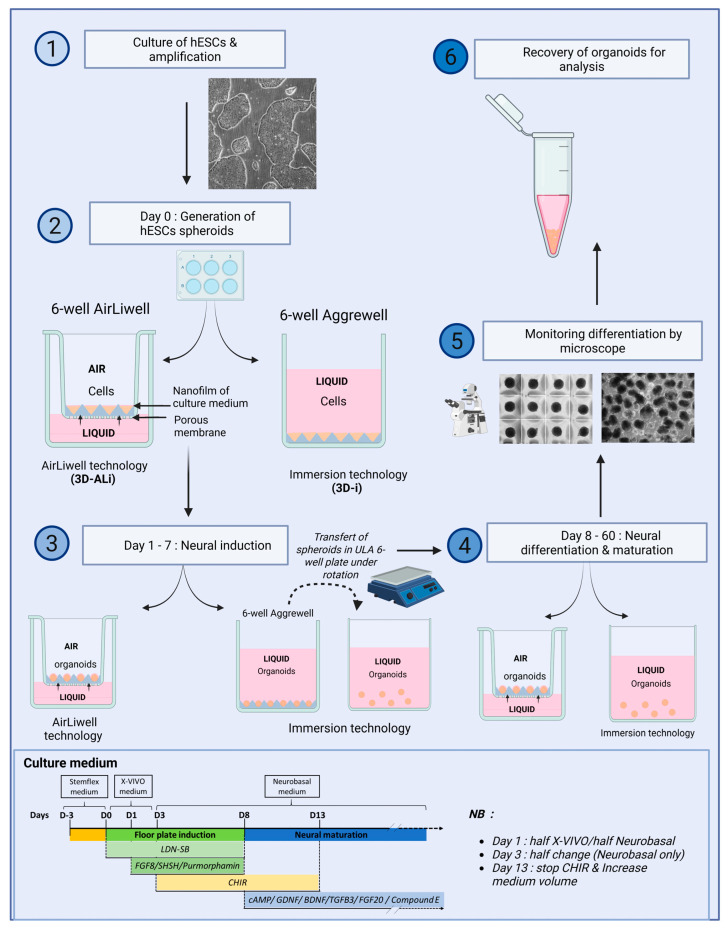
Graphical summary of the first part of material and methods regarding the generation and culture of human brain organoids (Created in Biorender. El Harane, S. (2025) https://BioRender.com/frr1adr).

HS420 cells were deposited in supplemented X-VIVO medium in Aggrewell-800TM (Stem cell technologies, 6-well plate, 2 mL per well) at the ratio of 2000 cells per microwell (the microwell plate used here contains 1600 microwells per well). To correctly distribute the cells in each microwell, the AggreWell plate was gently shaken and put on a stable support. After 15 min, the plate was cultured at 37 °C for 24 h to generate organoids. Next, 24 h after organoids formation, the spheres were collected and then transferred in a standard 6-well plate in a combination of half X-VIVO and half neurobasal medium without ROCK inhibitor supplemented with 1% of PenStep, 1% non-essential amino-acids, 1% B-27 supplement (Thermofisher), L-Glutamine, 0.5 μM of SB431542, 10 μM LDN193189 (dual-SMAD inhibition cocktail), 100 ng/mL of SHH, 2 µM of Purmorphamine, and 100 ng/mL FGF-8. Organoids were then cultured under constant agitation in 3 to 4 mL of medium per well, and cultured at 37 °C and 5% CO_2_ under constant agitation (60 rpm, orbital shaker). At day 3, 3 µM of CHIR99021 was added. The neurobasal medium progressively replaced the X-VIVO medium. At day 8, medium was replaced by neurobasal medium supplemented with 1% of PenStep, 1% non-essential amino-acids, 1% B-27 supplement, 3 µM of CHIR99021, 0.5 mM of cAMP, 20 ng/mL of GDNF, 20 ng/mL of BDNF, 5 ng/mL of FGF20, 1 ng/mL of TGF-B3, and 1 µM of Compound E. At day 13, CHIR99021 was removed. Importantly, for each medium exchange, the half of medium was refreshed, using between 1.5 and 2 mL per well (Scheme 1). Note also that with the immersion, organoids tend to fuse, which was not observed with the air–liquid interface organoids.

##### Generation and Culture of Midbrain Organoids by Using AirLiwell Technology (3D-ALi) [43]

For neural differentiation, 2000 human pluripotent stem cells per microwell were seeded in an AirLiwell plate (~800 microwells per well, in a 6-well plate). The AirLiwell technology, which was previously described [43], consists of individualized microwells made on a semi-permeable membrane, allowing culture at an air–liquid interface. This configuration optimizes nutrient and gas exchange while maintaining organoid separation. These plates are now commercially available internally at the University of Geneva and will soon be accessible to the broader scientific community. For more information regarding the technology and the availability of the plates, please contact sanae.elharane@unige.ch. In order to correctly distribute the cells in each microwell, the AirLiwell plate was gently shacked and put on a stable and flat support for 15 min. Supplemented X-VIVO medium to support their differentiation into neuronal lineages was added under the insert. Medium from the cell suspension added on the microwells was then removed by a 1000 μL pipet and organoids were just covered by a thin film of residual medium before incubation of the plate at 37 °C for 24 h (Scheme 1).

24 h after organoids formation, culture medium under the insert was replaced with fresh medium made by a combination of half X-VIVO and half neurobasal medium without ROCK inhibitor supplemented with 1% PenStep, 1% non-essential amino-acids, 1% B-27 supplement (Thermofisher), L-Glutamine, 0.5 μM of SB431542, 10 μM LDN193189 (dual-SMAD inhibition cocktail), 100 ng/mL of SHH, 2 µM of Purmorphamine, and 100 ng/mL FGF-8. Organoids were then cultured in their individualized microwells in a conventional incubator at 37 °C and 5% CO_2_. At day 3, 3 µM of CHIR99021 was added. The neurobasal medium replaced progressively the X-VIVO medium. At day 8, medium was replaced by neurobasal medium supplemented with 1% of PenStep, 1% non-essential amino-acids, 1% B-27 supplement, 3 µM of CHIR99021, 0.5 mM of cAMP, 20 ng/mL of GDNF, 20 ng/mL of BDNF, 5 ng/mL of FGF20, 1 ng/mL of TGF-B3, and 1 µM of Compound E. At day 13 CHIR99021 was removed: for each medium exchange, the half of medium was refreshed, using 0.5 mL per well (Scheme 1).

### 2.2. Comprehensive Content Analysis of Organoids (Scheme 2)

#### 2.2.1. Bulk RNA Sequencing: Sample Preparation

RNA was extracted from midbrain organoids cultivated in immersion (3D-i) and in air–liquid culture interface (3D-ALi) derived from 4 different cell batches of HS420 cells at week 6. Approximately 800 organoids were pooled for each bacj and each condition, and RNA was extracted from the pooled cells. Total RNA isolation was performed using the RNeasy kit (Qiagen, Hilden, Germany) according to the manufacturer’s instructions. RNA concentration was determined by a NanoDrop 2000 spectrometer (Thermofisher, Geneva, Switzerland) and RNA quality was verified by the 2100 Bioanalyzer (Agilent, Santa Clara, CA, USA).

#### 2.2.2. Bulk RNA-Sequencing Analysis

##### Library Preparation, Sequencing and Read Mapping to the Reference Genome

cDNA libraries were constructed by the Genomics Platform of the University of Geneva using the Illumina TruSeq RNA Sample Preparation Kit (Ilumina, San Diego, CA, USA) (check with the sequencing platform) according to the manufacturer’s protocol. Libraries were sequenced using single-end (100nt-long) sequencing on Illumina HiSeq2000 (Ilumina, San Diego, CA, USA) (check with the sequencing platform). FastQ reads were mapped to the ENSEMBL reference genome (GRCh38.96) using STAR version 2.4.0j [44] with standard settings, except that any reads mapping to more than one location in the genome (ambiguous reads) were discarded (m = 1). Sequence data have been submitted to the GEO database under the accession number.

##### Unique Gene Model Construction and Gene Coverage Reporting

A unique gene model was used to quantify reads per gene. Briefly, the model considers all annotated exons of all annotated protein coding isoforms of a gene to create a unique gene where the genomic region of all exons was considered coming from the same RNA molecule and merged together.

##### RNAseq Analysis

All reads overlapping the exons of each unique gene model were reported using featureCounts version 1.4.6-p1 [45]. Gene expressions were reported as raw counts and in parallel normalized in RPKM to filter out genes with a low expression value (1 RPKM) before calling for differentially expressed genes. Library size normalizations and differential gene expression calculations were performed using the package edgeR (version 3.28.0) [46] designed for the R software (version R-3.6.3) [47]. Only genes having a significant fold change (Benjamini–Hochberg corrected *p*-value < 0.05) were considered for the rest of the RNAseq analysis.

##### Gene Ontology and/or Pathways Analysis

GO term and pathways enrichment was performed using home-made scripts for the R software. A second gene ontology analysis was performed using gProfiler application considering the 50-most up- or down-regulated genes using the website (https://biit.cs.ut.ee/gprofiler/, accessed on July 2023).

##### GSEA: Gene Set/Pathway Enrichment Analysis

All annotated pathways for Homo sapiens, Mus musculus, Rattus norvegicus, Danio rerio, Sus scrofa, and Saccharomyces cerevisiae available on the WikiPathways database (http://www.wikipathways.org/index.php/WikiPathways, accessed on March 2023) were used to generate gene sets, as well as the KEGG (http://www.genome.jp/kegg/, accessed on March 2023) and Reactome (https://reactome.org/, accessed on March 2023) pathways relative to GRCh38.96. Genes were ranked by their calculated fold changes (decreasing ranking). A gene set analysis using the GSEA package Version 2.2 [48,49] from the Broad Institute (MIT, Cambridge, MA, USA) was used to analyze the pattern of differential gene expression between the two groups. Gene set permutations were performed 1000 times for each analysis. The normalized enrichment score (NES) was calculated for each gene set. GSEA results with a nominal FDR < 0.05 and abs (NES) > 1 were considered significant.

##### De Novo Motif Discovery and Motif Enrichment Analysis

Promoter sequences (−1000 bp to +500 bp relative to the TSS) were extracted, oriented according to gene orientation, and used as input for de novo motif discovery using the cosmo package (version 1.20.0) [50] designed for R. The probabilistic model used for the motif discovery is the zero-or-one-occurrence-per-sequence (ZOOPS), considering only the orientation of each promoter and motif lengths between 10nt and 20nt. Pscan [51] was used to identify potential TF-binding-sites (TFBSs) that were overrepresented in each promoter category, using the JASPAR database [52] as matrices. TFBSs with *p*-values lower than 0.001 were considered to be significantly overrepresented.

#### 2.2.3. Quantitative RT PCR

For qRT-PCR, RNA was extracted from midbrain organoids cultivated in immersion (3D-i) and in air–liquid culture interface (3D-ALi) derived from 4 different cell batches of HS420 cells. The time points for RNA extraction were week 4 and 6. Total RNA isolation was performed using the Qiagen RNeasy kit according to the manufacturer’s instructions. RNA concentration was determined by a spectrometer (Thermo ScientificTM NanoDrop 2000) and RNA quality was verified by the 2100 Bioanalyzer (Agilent, Santa Clara, CA USA). Quantitative PCR was performed with Tecan Freedom Evo by using SYBR green fluorescence. cDNA was produced from RNA by using the prime script RT reagent kit (Takara). All primers (Table 1) were diluted at a concentration of 0.86 μM, and a master mix was used (Power SYBR Green Master Mix, Thermo Fisher Scientific).

#### 2.2.4. Sample Preparation for Single-Cell RNA Sequencing

Samples containing approximatively 800 organoids coming from immersion culture or from air–liquid interface culture were dissociated using a neural tissue dissociation kit from Miltenyi according to the manufacturer’s instructions. Dissociated cells were then sorted by flow cytometry using double staining. Draq7 was used to identify and eliminate dead cells and Hoechst was used to distinguish between cells and cellular debris. Viable cells were sorted and directly transferred to the Genomics Platform of Geneva to process the samples.

#### 2.2.5. Single-Cell RNA Sequencing Analysis

The analysis was performed independently and in a double-blinded setting.

Samples were processed on the 10x Genomics Chromium system. The Chromium Next GEM Single Cell 3′ v3.1 and Dual Index TT kits (10x Genomics, Pleasanton, CA, USA) were used for library preparation according to manufacturer’s specifications. Libraries were sequenced on HiSeq 4000 (Ilumina, San Diego, CA, USA) for a depth of around 100 million reads per sample.

Cell and UMI barcodes were identified with UMItools (version 1.0.1) [53], expecting 10,000 cells per sample.

FastQ reads were mapped to the ENSEMBL reference genome (GRCh38.96) using STAR version 2.7.1a [44] with standard settings, except that any reads mapping to more than one location in the genome (ambiguous reads) were discarded (m = 1). A unique gene model was used to quantify reads per gene. Briefly, the model considers all annotated exons of all annotated protein coding isoforms of a gene to create a unique gene where the genomic region of all exons is considered coming from the same RNA molecule and merged together.

All reads overlapping the exons of each unique gene model were reported using featureCounts version 1.6.4 [45]. UMIs per gene per cell were counted using UMItools and imported in Seurat version 3.1.2 [54] designed for R [47]. Cells were sorted according to their number of detected genes in order to exclude doublet/multiplet as well as their mitochondrial content, using the subset() function.

Datasets were integrated following the recommendations of the Seurat development team [55]. Cell types were identified using the Mouse Cell Atlas [56] and the seven references available in the celldex package for R (https://rdrr.io/github/LTLA/celldex/). Mouse references were humanized using biomaRt R package (https://bioconductor.org/packages/release/bioc/html/biomaRt.html). From the calculated prediction scores, we used the five highest-scoring cell types per cluster to guide the annotation. Markers of each cluster were identified using the FindConservedMarkers() function whereas differentially expressed genes between datasets (in each previously identified clusters) were identified using the FindMarkers() function.

#### 2.2.6. Midbrain Organoids Plating on Extracellular Matrix, Immunocytochemistry, and Electron Microscopy

After 1 month of differentiation, the midbrain organoids were plated on polyornithine/laminin-coated tissue culture plates in maturation neurobasal medium supplemented with 1% of penicillin/streptomycin, 1% non-essential amino-acids, 1% B-27 supplement, 3 µM of CHIR99021, 0.5 mM of cAMP, 20 ng/mL of GDNF, 20 ng/mL of BDNF, 5 ng/mL of FGF20, 1 ng/mL of TGF-B3, and 1 µM of Compound E. After 2 weeks of culture on coverslips, cells on glass coverslips were fixed with 1 mL of 4% paraformaldehyde in PBS for 30 min at room temperature. Cells were then washed three times with 2 mL of PBS. Cells were permeabilized with PBS containing 0.3% Triton X-100 (Sigma-Aldrich, Saint-Louis, MO, USA) and incubated overnight with following primary antibodies in PBS containing 5% bovine serum-albumin (Sigma), mouse anti- βIII-tubulin (Sigma), and rabbit polyclonal anti-TH (Merck, Darmstadt, Germany).

Cells were washed three times with 2 mL of PBS before incubation for 1 h 30 min with the secondary antibody (goat-anti-mouse IgG—Alexa 555 (Thermofisher, Geneva, Switzerland), goat anti-rabbit IgG–Alexa 488 (Life technologies, Carlsbad, CA, USA)) in PBS containing 5% bovine serum-albumin. Cells were washed three times with PBS before exposure to DAPI 300 nM in PBS (Sigma) for 15 min at room temperature. After three washes in PBS, cells were rinsed with water and mounted in glass slides using Fluorsave reagent (Millipore, Burlington, VT, USA). Moreover, midbrain organoids plated on the glass coverslips were dehydrated, then covered with a gold nanolayer (20 nm) and visualized by scanning electronic microscopy.

For Hematoxylin–Eosin (H&E) staining, organoid samples were fixed in 4% paraformaldehyde in PBS, followed by an inclusion of organoids in a drop of agar and dehydration through a graded ethanol series. The fixed organoids were then embedded in paraffin, sectioned at 5 µm, and mounted on glass slides. Sections were deparaffinized, rehydrated through a descending ethanol series, and stained with Hematoxylin for 5 min, followed by a 2 min counterstaining with Eosin. After washing with water and dehydrating through ethanol, the slides were cleared and mounted with a coverslip using a mounting medium.

Immunostaining was also performed on paraffin-embedded organoid sections. After deparaffinization and rehydration, antigen retrieval was performed by incubating the sections in citrate buffer (pH 6.0) at 95 °C for 15 min. Following cooling to room temperature, sections were blocked with 5% bovine serum albumin in PBS, followed by incubation overnight with primary antibodies specific to markers TH (ab152, Merck), NURR1 (ab60149, Abcam, Cambridge, UK), LMX1A (ab139726, Abcam), FOXA2 (IC2400G), TUBB3 (T8660, Sigma), MAP2 (M4403, Sigma), DDC (AB1569, Merck), KI67 (ab16667, Abcam), GFAP (MAB360, Merck), and cleaved-caspase 3 (9661S, Cell signaling, Danvers, MA, USA). After incubation with appropriate secondary for 30 min (except for the FOXA2 antibody which was already conjugated), sections were counterstained with Hoechst and mounted on glass slides with Fluorsave reagent (Millipore, Burlington, VT, USA).

#### 2.2.7. Electrophysiology by Micro-Electrode Array (MEA)

Midbrain organoids were transferred to the center of a porous multi-electrode array (MEA) device using a dissection microscope, as previously described [57]. For each experimental condition, electrical activity was recorded using 8 electrodes, with each condition tested in triplicate. The recordings were performed using an amplifier and a data acquisition system, with no external stimulation applied, capturing only spontaneous neuronal activity. The signal-to-noise ratio (SNR) was calculated as the standard deviation of the voltage during a 30 min recording period, with the signal being defined as the average peak-to-peak voltage of the recorded spikes. Recordings were conducted over a 50-day period, following 4 months (at day 123) of organoid maintenance, to allow for sufficient neuronal maturation as recommended by our collaborators for reliable functional activity detection.

#### 2.2.8. Dopamine Dosage by HPLC

Dopamine production was analyzed by high-pressure liquid chromatography (HPLC) after extraction from midbrain organoids cultivated in immersion (3D-i) or in air–liquid culture interface (3D-ALi) in 0.1 N perchloric acid (HClO4). Cells were lysed in a small volume (250 uL) for 15 min at 4 °C with vigorous vortexing every 5 min. Supernatant was recovered after centrifugation and used for dopamine dosage immediately or stored at −20 °C. Catecholamines were measured by HPLC with electrochemical detection in coulometric mode. Separation of the analytes was achieved on a reversed-phase column, Symmetry C-18, Kinetex 5μ C18 100A° (250 × 4.6 mm) (Phénomenex, Torance, CA, USA), in isocratic mode at a flow rate of 0.8 mL/min. The coulometric detector parameters, ECD-000RS (Thermo scientific, Geneva, Switzerland), were for conditioning cells at a potential of +300 mV. For analytical cells, an electrode 2 at a potential +50 mV and electrode 3 at a potential of −300 mV weare used.

#### 2.2.9. Statistical Analyses

Statistical analysis was performed using GraphPad Prism v.10.2.3. For comparisons between groups, non-parametric t-tests were conducted. Data are presented as mean ± standard deviation (SD). Statistical significance was determined with *p*-values indicated as follows: * *p* < 0.05, ** *p* < 0.01, *** *p* < 0.001, **** *p* < 0.0001. All statistical tests were performed based on the assumptions of the data, and appropriate methods were selected to ensure accurate interpretation of the results. Statistical analysis for bulk and single-cell RNA sequencing data was performed using R. Detailed information of the specific methods used for these analyses can be found in the respective section of Section 2.

## 3. Results

### 3.1. Midbrain Organoids Generated at Air–Liquid Interface Are Highly Standardized

In the standard method immersion (3D-i) (Figure 1A), organoids were first force-aggregated in plastic microwells, then transferred in standard culture plates and maintained under agitation. With the AirLiwell method (3D-Ali) (Figure 1B), organoids were forced to aggregate in non-adhesive microwells and individually maintained within these microwells without the need for transfer or agitation. A semi-permeable membrane at the bottom of the organoids allows for their long-term stability and also establishes air/liquid interface conditions that favor gas exchanges with air. To compare and assess the precise morphology of midbrain organoids in immersion (3D-i) versus in air–liquid interface (3D-ALi), a dopaminergic-oriented midbrain differentiation for 2 months was performed using a standard protocol (Figure 1C, [28]). Homogeneity of midbrain organoids was studied over 60 days. The evolution of their size and shape was followed by light microscopy.

As shown in Figure 1D, there was no significant difference in both the round shape and size between the two methods in week 0. However, after 2 weeks of culture, 3D-ALi organoids were more homogeneous than those grown on immersion. A software-based quantification of the range of the distributions of the area, diameter, and roundness (Figure 1E) showed a clear lower variability of 3D-ALi organoids. In addition, they kept their round morphology while those 3D-I organoids evolved into more heterogeneous and irregular shapes.

### 3.2. Bulk RNA Sequencing Confirmed and Showed a Better Neural/Midbrain Specification

**Figure 2:**
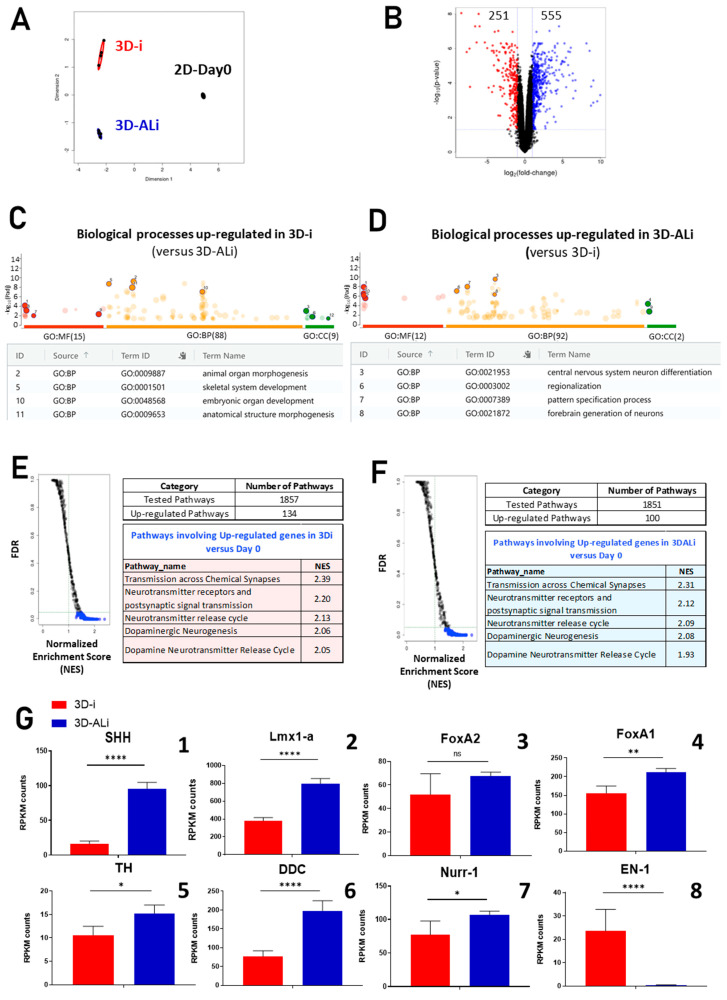
Validation of enhanced neural and midbrain specification through bulk RNA sequencing (n = 4; after 6 weeks of differentiation): (**A**) multidimensional scaling plot; (**B**) volcano plot depicting regulated genes in 3D-Ali organoids compared to 3D-i organoids; (**C**) gene ontology analysis results, with a table and representation of up-regulated biological processes, cellular components, and molecular functions in 3D-i organoids and (**D**) in 3D-ALi organoids; (**E**) gene set enrichment analysis (GSEA) illustrating the number of regulated pathways compared to pluripotent stem cells at day 0, including the top 15 most up-regulated pathways with their respective normalized enrichment scores (NES) in both immersion and (**F**) air–liquid interface organoids; (**G**) graphs displaying gene expression (in RPKM) of genes involved in dopaminergic neurogenesis and late dopaminergic differentiation, with statistical significance (*p*-Value = *p*) indicated as follows: ns *p* >0.05 non-significant, * *p* < 0.05, ** *p* < 0.01, **** *p* < 0.0001 (EDGE R analysis).

To have a global view about gene expression and to study midbrain specification, a bulk RNA-sequencing analysis was performed to compare organoids cultivated by both methods at week 6 of differentiation (n = 4). A control made of cells in 2D and at day 0 was also included. A multidimensional scaling analysis (MDS) in Figure 2A showed three different and distinct populations with closer quadruplicates for 2D- and 3D-ALi conditions. At week 6 of cell culture, the cells cultivated in both 3D cell culture methods had a strongly different gene expression profile. We observed that 555 genes were up-regulated and 251 down-regulated in 3D-ALi culture compared to 3D-i condition (Figure 2B). To characterize and understand these gene expression differences, we carried out a gene ontology analysis on the 50 most up-regulated genes comparing 3D-i and 3D-ALi organoids (Supplementary Table S1). We found that for immersion organoids, most up-regulated genes are related to biological processes of animal organ morphogenesis, skeletal systems, embryonic organ development, and anatomical structure morphogenesis (Figure 2C). While for 3D-ALi organoids, biological processes which were overrepresented were related to “central nervous system neuron differentiation”, “regionalization and pattern specification” processes, as well as “forebrain generation of neurons” processes (Figure 2D). Considering the cellular components that were represented, for both conditions, we found chromatin; however, for 3D-i organoids, “extracellular matrix” as well as “external encapsulating structure” were also more represented. Finally, we did not observe differences in molecular functions, and we found for both conditions the presence of “DNA-binding transcription factor activity” and “double-stranded DNA binding”, except for “extracellular matrix structural constituent” which was only represented in immersion organoids.

During both types of 3D differentiation (immersion vs. air–liquid interface), an up-regulation of genes involved in dopaminergic neurogenesis and dopamine neurotransmitter release cycle was observed (Figure 2E,F). For the pathway “dopaminergic neurogenesis”, the NES was slightly higher in 3D-ALi organoids in comparison to immersion organoids, while for the pathway “dopamine neurotransmitter release cycle”, the NES was higher in 3D-i organoids. Among the most up-regulated pathways, we also found in both conditions pathways that were related to “transmission across chemical synapses”, “neurotransmitter receptors and postsynaptic signal transmission”, and “neurotransmitter release cycle”.

Then, we more precisely assessed midbrain markers involved in DA neuron development and differentiation (Figure 2G). We found that Sonic Hedgehog (*SHH*) which play a critical role in development of dopaminergic neurons (DA) during early embryonic stages was highly expressed in 3D-ALi in comparison to 3D-I organoids. The expression of *LMX1A* known to play a pivotal role in the DA differentiation of human pluripotent stem cells 50 was significantly more expressed in 3D-ALi organoids. FoxA1 and *FOXA2*, two transcription factors known to be crucial for the specification of DA neurons and also for survival of DA neurons [58,59,60], were also up-regulated in 3D-ALi organoids.

We also evaluated the gene expression level of Tyrosine Hydroxylase (*TH*) and dopa decarboxylase (*DDC*), two important enzymes involved into DA metabolism and specification of DA neurons [61], and we observed that they were significantly more expressed in 3D-ALi organoids in comparison to the standard 3D-i organoids. We also studied the expression of Nurr-1, a member of the nuclear receptor superfamily of transcription factors which was expressed predominantly in the central nervous system, particularly in developing and mature dopaminergic neurons and we also observed higher gene expression in 3D-ALi organoids [62]. Others genes involved in dopaminergic early development or late differentiation also showed a significant increase in gene expression (*BARHL1*, *ASCL1*, *NTN1*) and an increased tendency (*OTX2*, *MSX-1*) in 3D-ALi organoids (Supplementary Figure S1A). However, the gene expression of Engrailed-1 (*EN-1*), which is also known to be involved in the development and survival of DA neurons, was significantly higher in 3D-i organoids [63,64]. We also observed that *DRD2*, also specific to mature dopaminergic neurons, was significantly up-regulated in 3D-i organoids (Supplementary Figure S1A). Furthermore, the evaluation of general neuronal maturation markers showed a significant up-regulation of *MAP2*, *NEFL* (Neurofilament light), *NEFM* (Neurofilament medium), *NEUN* and *SYP* (synaptophysin) in 3D-i organoids (Supplementary Figure S1B). No significant difference was observed for *NEFH* (Neurofilament heavy), *PSD-95*, and *ENO2*, also known to be expressed in mature neurons.

In conclusion, the bulk RNA-sequencing analysis comparing organoids cultivated using the air–liquid interface and immersion methods has provided valuable insights into the gene expression profiles and biological processes associated with dopaminergic specification. The results demonstrate distinct patterns of gene expression between the two methods, with significant differences observed at week 6 of cell culture. The 3D-ALi organoids exhibiting up-regulation of genes associated with dopaminergic neuron development and differentiation, including *SHH*, *LMX1A*, *FOXA1*, *FOXA2*, *TH*, *DDC*, and *NURR1*, were found to be significantly higher in 3D-ALi organoids compared to immersion organoids. The 3D-i organoids showed a higher expression of genes related to neuronal maturity.

### 3.3. Histological Comparison Reveals Enhanced Homogeneity in 3D-ALi Organoids

**Figure 3:**
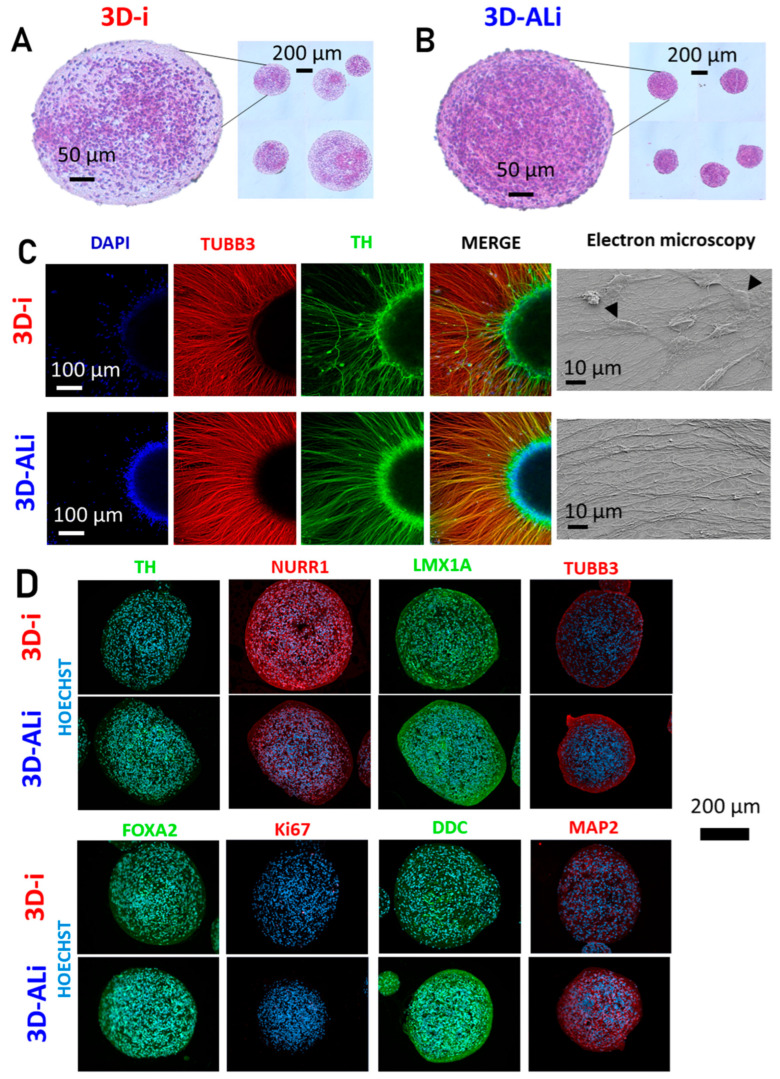
Histological comparison reveals enhanced homogeneity in 3D-ALi organoids: (**A**) Hematoxylin–Eosin colorations of organoid sections from 3D-i organoids, and (**B**) 3D-ALi organoids at 6 weeks of differentiation. (**C**) Neuronal maturation of midbrain organoids on poly-ornithine laminin observed in immersion (**upper side**) and in an air–liquid culture interface (**lower side**) using immunofluorescence with Anti-TUBB3 (red)/TH (green) and DAPI (blue) staining after 6 weeks of differentiation (including 2 weeks of culture on poly-ornithine laminin). On the right: scanning electron microscopy images showing neuronal networks in both 3D-i and 3D-ALi organoids, and large flattened cells (indicated by black arrows) specifically observed in 3D-i organoids. (**D**) Immunostaining of organoid sections after 6 weeks of differentiation showing the expression of the following markers: TH (green), NURR1 (red), LMX1A (green), TUBB3 (red), FOXA2 (green), KI67 (red), DDC (green), and MAP2 (red). All nuclei were stained with Hoechst (blue). The respective scale bars are indicated in each image.

In order to characterize histological aspects and cell features inside organoids, we first performed some sectioning on organoids. Coloration using Hematoxylin–Eosin of organoids sections showed a different cell repartition in organoids cultivated in immersion (3D-i) or at air–liquid interface (3D-ALi). Indeed, in the outer part of the 3D-i organoids, we observed a low density of cells (Figure 3A), while in the 3D-ALi organoids, the cells were distributed homogeneously even in the outer part of the organoid with a comparable cell density in all organoid regions (Figure 3B). To evaluate the neurite outgrowth, we performed neuronal maturation of organoids by their plating on extracellular matrix-coated (ECM) plates at week 4 of differentiation. After 1 week of maturation, an immunofluorescence staining was performed with the neuronal marker beta-III tubulin and TH, a specific marker of dopaminergic neurons (Figure 3C). Abundant neurons were obtained around plated spheres with both methods and no significant difference was observed (Supplementary Figure S3A). The mature neurons on ECM-coated plates were also observed by electron microscopy (Figure 3C-right and Supplementary Figure S6A). This allowed for the analysis of the neuronal network and the shape of the cells with a better resolution. For both conditions, a dense neuronal network was observed. However, in the immersion condition, the presence of invading large flattened cells (black arrow) was observed underlying the neuronal network. In contrast, the air–liquid interface method did not induce this cell population, showing a higher level of homogeneity and purity.

We next performed immunofluorescence analysis on sections of 3D-i and 3D-ALi midbrain organoids (Figure 3D and Supplementary Figure S2C). Visual inspection suggested that 3D-ALi organoids had more cells positive for TH, DDC, and FOXA2, possibly indicating an enrichment in dopaminergic lineage markers. For other markers, including MAP2, GFAP, TUBB3, NURR1, and LMX1A, no clear difference in the number of positive cells was observed between conditions. We next performed quantification of fluorescence intensity using ImageJ software (version 1.54p). Note that this analysis measures total mean fluorescence within organoids sections and not the percentage of positive cells. The results showed comparable expression levels of MAP2 and TH in both types of organoids, a non-significant trend toward higher expression for TUBB3, DDC, GFAP, and FOXA2 in 3D-ALi organoids, and a trend towards increased expression of NURR1 and LMX1A in 3D-i organoids. Both conditions showed very low or absent expression of Ki67 and cleaved caspase-3, suggesting low proliferative activity and absent or minimal apoptosis at the time of analysis.

### 3.4. Single Cell Analysis of Organoids Showed a Significantly Higher Yields of Neuronal Cells

**Figure 4:**
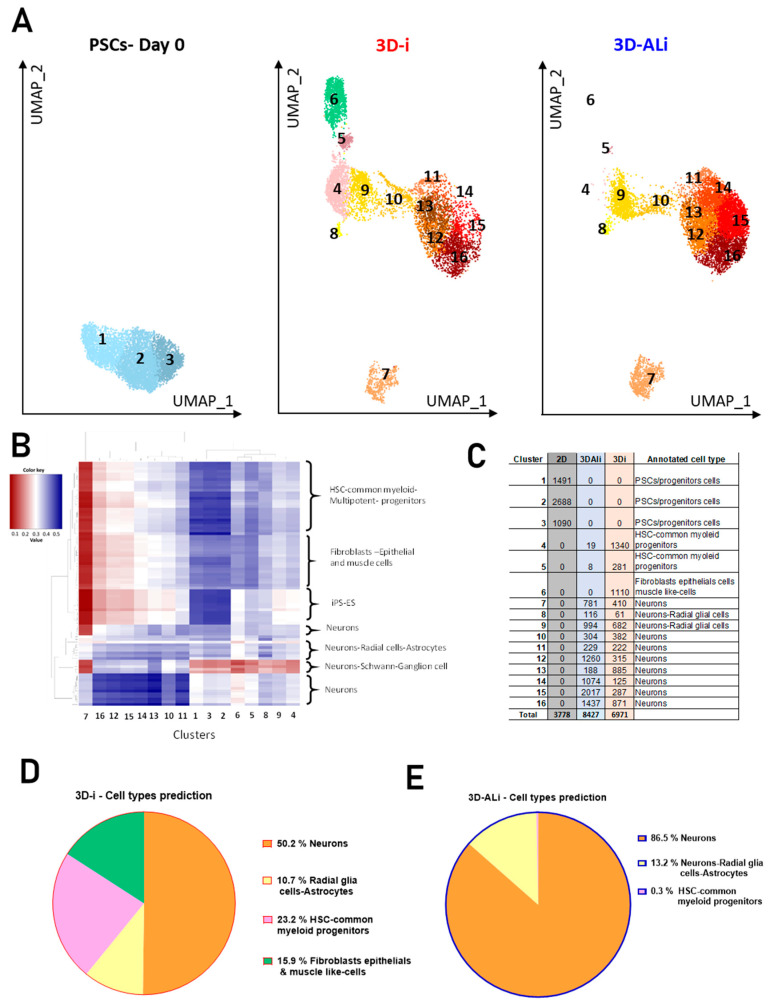
3D-ALi organoids contain a high yield of neural cells: (**A**) UMAPs showing different clusters present in 2D cells at Day 0, in 3D-i organoids, and in 3D-ALi organoids after 6 weeks of differentiation (from left to right); (**B**) heatmap displaying cell type annotation scores, with each row representing a reference cell subtype and each column representing a cluster from our dataset. Scores are color-coded: positive scores are shown in shades of blue, and negative scores in shades of red. Darker colors indicate stronger associations—either positive or negative—between clusters and cell subtypes. (**C**) Table showing the number of cells per cluster across all conditions, along with their corresponding annotated cell types. (**D**) Pie-chart showing the proportion of different cell types found in 3D-i organoids; (**E**) pie-chart showing the proportion of different cell types found in 3D-ALi organoids. (Orange color: neurons, yellow color: neural progenitors, radial glia cells and astrocytes, pink color: HSC and common myeloid multipotent progenitors, green color: fibroblasts, epithelial and muscle like-cells).

To understand the nature of these invading cells and to precisely characterize the content of the organoids in terms of cell types and differentiation, we carried out single-cell RNA sequencing (sc-RNA seq). For this, we analyzed organoids at 6 weeks of differentiation generated by the two methods as well as pluripotent cells (PSCs) initially used at day 0. Under these conditions, 16 cell clusters were identified (Figure 4A). Pluripotent cells were grouped into three close clusters (PSCs: Day 0, Figure 4A). Brain organoids generated by the immersion and air–liquid interface methods were clustered into 13 and 12 clusters, respectively (Figure 4A). There was no overlap in cell clusters between the brain organoids and the pluripotent stem cells (1, 2 and 3).

The clusters found in the organoids produced by immersion (3D-i) were also found in the organoids produced by the air–liquid interface method (3D-ALi), except for cluster 6, which was not present in 3D-ALil organoids. We then studied in detail the composition and proportion of cells in each cluster (Figure 4B,C). Clusters 4, 5, and 13 were more abundant in the 3D-i condition as compared to the 3D-ALi condition. Inversely, clusters 7, 8, 9, 12, 14, 15, and 16 contained more cells in the 3D-ALi condition than in the 3D-i condition (Figure 4C). For other clusters, such as 10 and 11, no difference in cell number distribution was observed between organoids produced by both methods (Figure 4C).

Then, to better understand the differences and identify the cell types contained in each cluster, a prediction of cell types was made by comparing the organoid cells with referenced human cell types and by calculating a score (see material and methods). A heatmap shows these results (Figure 4B). The detected cell types could be classified the into six subfamilies, such as HSC-common myeloid and multipotent progenitors, fibroblasts/epithelial, and muscle cells, pluripotent stem cells (iPSs-ESCs), neurons, neurons/radial cells, and astrocytes, as well as neurons/Schwann cells. These results showed that clusters 1, 2, and 3 found in the pluripotent stem cells at day 0 presented a high prediction score for pluripotent stem cells but also for hematopoietic stem cells, myeloid, and multipotent progenitors. Cluster 4 and 5 mainly found in 3D-i organoids seems to be also related to HSC-common myeloid and multipotent progenitors. Cluster 6, which was only found in immersion organoids seems to be composed of fibroblasts/epithelial and muscle-like cells. Clusters 8 and 9 showed a high score for neurons/radial cells and astrocyte cell types. Finally, all the rest of the clusters including cluster 7, 10, 11, 12, 13, 14, 15, and 16 were neuronal cells based on the prediction score. From this analysis and from the number of cells per cluster, we could calculate precise repartition of cells contained within organoids cultivated in immersion and at the air–liquid interface.

We observed that 3D-i organoids were composed of 50.2% of neurons, 10.7% of neurons/radial glial cells and astrocytes, 23.2% of common multipotent progenitors, and of 15.9% of fibroblast-like cells (Figure 4D), while 3D-ALi organoids were composed of 86.5% of neurons, 13.2% of neurons/radial glial cells, and only 0.3% of common multipotent progenitors (Figure 4E). This showed that 3D-ALi organoids gave significantly higher yields of neurons and much less contaminant or unwanted cell types. A second independent analysis, based on data from the Mouse Allen Brain Atlas, also confirmed the same distribution of neuronal and non-neuronal cells observed in organoid cultures. This analysis further supports our findings, demonstrating consistent patterns in cell distribution across both analyses approaches (Supplementary Figure S2A). The expression of key midbrain dopaminergic markers including *LMX1A*, *FOXA2*, *NURR1* (*NR4A2*), and *TH* was detected across clusters 9 to 16 (Supplementary Figure S5). This distribution supports the presence of cells with midbrain identity within these clusters and likely reflects the heterogeneity and dynamic states of differentiation within the midbrain dopaminergic lineage, which span early progenitors to more mature neuron-like cells. This distribution supports the presence of cells with midbrain identity within these clusters and likely reflects the heterogeneous and dynamic nature of dopaminergic differentiation, spanning early progenitor stages to more mature neuron-like states. We also studied the cell cycle using these sc-RNAseq data, and we observed that pluripotent stem cells at day 0 were mainly in S and G2M phases, while cells contained in organoids cultivated by both techniques were in majority in phase G1, with a low number of cells in phase S and G2M. No significant difference with respect to cell cycle was observed between 3D-ALi and 3D-i organoids (Supplementary Figure S2B).

### 3.5. 3D-ALi Organoids Are Electrically Active and Showed a Higher Degree of Synchronization

**Figure 5:**
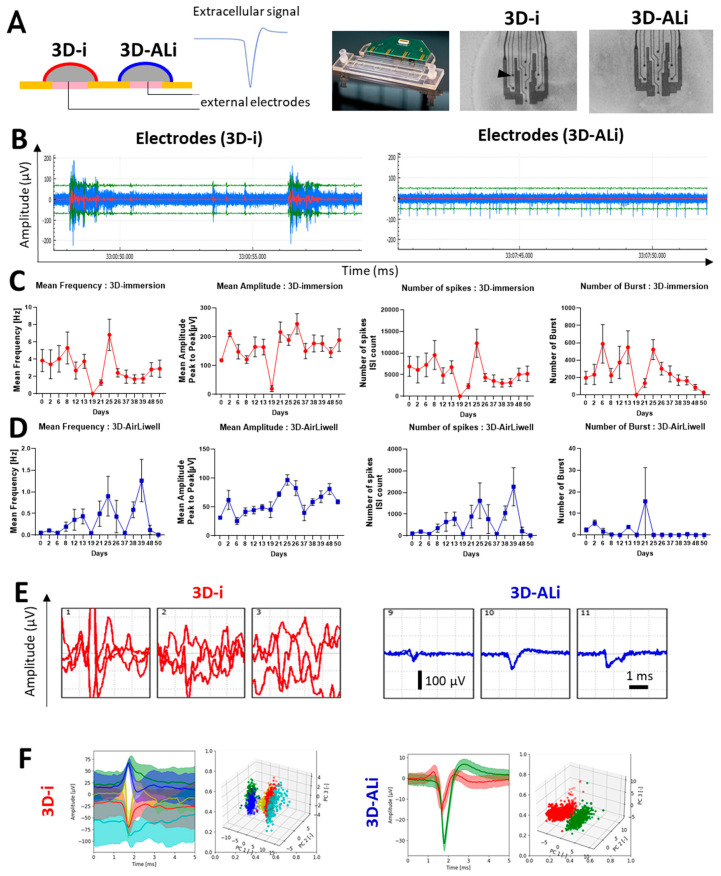
3D-ALi organoids showed a high degree of synchronization of their electrical signal: (**A**) (From left to right): Schematic illustration of the experimental procedure, the theoretical expected extracellular signal, picture of the microelectrode Array chip used in this experiment, microscopy picture of 3D-i organoids under the electrodes (shown with small black arrow on right), microscopy picture of 3D-ALi organoids under the electrodes; (**B**) raw recording of the electrical signal in immersion organoids (**left**) and in air–liquid interface organoids after 4 months of differentiation (**right**); (**C**) from left to right, graphs showing the time course (50 days) measurement of mean frequency of electrical activity, mean amplitude, number of spikes, and number of bursts in 3D-i organoids and; (**D**) in 3D-ALi organoid; (**E**) pictures of the electrical signal over time and recorded on 3 independent electrodes in 3D-i organoids (**on left**) and 3D-ALi organoids (**on right**); (**F**) principal component analysis graph, showing spike sorting in 3D-i (**left**) and in 3D-ALi organoids (**right**). (Each point represents a spike. Each color represents a cluster).

To study the functionality of organoids, we studied their electro-physiological activity. The spontaneous electrical activity generated by the organoids differentiated by the two techniques was measured through the extracellular signal by microelectrode array (MEA) (Figure 5A). First, electrical activity could be observed for both organoids cultivated in immersion and at the air–liquid interface, showing that cells were alive and electro-physiologically active (Figure 5B). However, 3D-i organoids showed a more intense electrical activity (Figure 5B). We then analyzed the electrical activity over 50 days, by the study of the mean frequency, mean amplitude, number of spikes, and number of burst of the electrical signal. During these 50 days, we were able to observe an electrical activity with some variations for both organoids produced in immersion and in the air–liquid interface (Figure 5C,D). Then, we analyzed the average of the mean frequency in both conditions during these 50 days and we observed that the mean frequency of 3D-i organoids was significantly higher than 3D-ALi organoids (Supplementary Figure S7A). The mean amplitude also showed the same results; 3D-i organoids have a mean amplitude of 165.1 µV increasing up to 300 µV at some time points while 3D-ALi organoids have a mean amplitude of 58.1 µV increasing up to 100 µV. We observed that the number of spikes was also significantly higher in 3D-i organoids (Supplementary Figure S7A). We also studied the presence of burst which was defined by a pattern of activity of multiple spikes in the same period of time and we observed that these phenomena were frequently observed in 3D-i organoids.

After a quantitative analysis, we assessed the qualitative features of the electrical signal by analyzing the waveform of the electrical signal detected in 3D-i and 3D-ALi organoids. The spike waveforms recorded from three different electrodes at the same time showed heterogeneous electrical signals in 3D-i organoids, whereas the 3D-ALi organoids exhibited more homogeneous waveforms, closely resembling the expected extracellular signal (Figure 5E). This increased homogeneity was consistently observed when analyzing electrical signals across eight different electrodes (Supplementary Figure S7B). Then, we performed a spike sorting which was used to separate the different waveforms that we can observe in the electrical signal. The spike sorting uses the principal component analysis technique to cluster the different waveforms (shape) of electrical signals. The spatial position of one spike was representative of its waveform (shape). The spikes with the more similar waveform were grouped by clusters using a different and unique color. After performing the spike sorting, we observed that the waveform of 3D-i organoids, the shape was more heterogeneous and grouped into more than four different shapes (Figure 5F) while the signal of 3D-ALi organoids was more homogeneous and grouped into two similar shapes (Figure 5F). In conclusion, brain organoids cultivated in immersion showed significant higher frequency, amplitude of spikes and burst, and a higher number of spikes and number of bursts. However, organoids grown at the air–liquid interface show calm and stable activity over time (Figure 5). Thus, 3D-ALi organoids appeared to have a more synchronized electrical activity than 3D-i organoids.

## 4. Discussion

This study introduces a new method for generating midbrain organoids. Present standard methods rely on culture of immersed organoids in suspension [27,28]. This approach is unreliable, as it requires constant agitation, may lead to organoid fusion [29,30,31,32], and exposes organoids to shear stress [29,33] and hypoxia [34,36,37,38,65]. Air–liquid interface cultures have shown promise for neuronal survival and axonal growth [39,40,66]. Yet, presently available air–liquid interface cultures are not compatible with a scalable organoid production that meets industry and clinical standards [42]. In this study, we have developed a novel approach, termed AirLiwell, that resolves these challenges by maintaining thousands of organoids in microwells without need for agitation, preventing organoid fusion, sheer stress, and hypoxia (Supplementary Figure S1C). It lowers costs through a low volume of culture medium and improves organoid standardization in size and shape.

The differences in cellular composition of immersion (3D-i) vs. air–liquid interface (3D-ALi) organoids, as assessed by single-cell RNAseq (sc-RNAseq), were striking. The 3D-i organoids contained fibroblast-like cells (16%), as well as cells of hematopoietic origin (23%) in addition to neural cells (61%). This was also suggested by electron microscopy, which detected fibroblast-like cells within the neuronal network. In contrast, 3D-ALi organoids consisted of 99% of neural cells. Such differences were also substantiated by gene ontology and gene set enrichment analysis. The 3D-i organoids predominantly expressed genes associated with organ formation and skeletal development suggesting that this type of differentiation does not exclusively induce neurogenesis. In contrast, 3D-ALi organoids predominantly expressed genes associated with neuronal differentiation in the central nervous system. Thus, scRNAseq suggested a larger percentage of neuronal cells in 3D-ALi organoids. The data on markers of mature neurons were slightly conflicting. Bulk RNAseq suggested that—as compared to 3D-i organoids—mRNA levels of markers of neuronal maturity (e.g., *MAP2*, *Neun* etc.) were moderately decreased in 3D-ALi organoids. However, this was not observed at the protein level, where MAP2 and TUBB3 appeared slightly increased in 3D-ALi organoids but without statistical significance. The following markers of midbrain/dopaminergic specification were enhanced 3D-ALi in both bulk (Figure 2) and scRNAseq (Supplementary Figure S5): *LMX1A*, *FOXA2*, *NURR1*, *DDC*, and *TH* as compared to immersion organoids. The quantitative analysis of the immunofluorescence data did not show any statistically significant difference between the two types of organoids. During our analysis of organoid morphology, we consistently observed the formation of rosette-like structures across both the immersion and air–liquid interface (ALI) culture methods. These rosettes are indicative of early neuroepithelial organization, a hallmark of neural differentiation. However, these structures did not progress into defined ventricle-like luminal compartments, as previously reported in midbrain organoids by Jo et al. [3]. This difference may reflect variations in protocol design, timing, or other intrinsic factors influencing tissue morphogenesis.

From a mechanistic point of view, the following working hypotheses might explain the differences in purity and maturation between the two types of midbrain organoids. The low oxygen supply in immersion organoids may interfere with neural induction. Indeed, it has been shown that hypoxia inhibits early steps of neural differentiation in embryonic stem cells [67]. Of particular interest was the high-level expression of the transcription factor *SHH* in 3D-ALi organoids. The SHH pathway is essential for early dopaminergic specification, leading to ventralization, and therefore, to the induction of *LMX1A*+/*FOXA2*+/*OTX2*+ ventral midbrain floor plate precursors. In line with this mechanism, we observed high expression levels of the three latter genes in 3D-ALi organoids.

The 3D-i organoids displayed a greater variability with respect to size, shape, and electrical activity. We hypothesize that the size differences of immersion organoids affect their nutrient and oxygen access, causing hypoxic regions to a variable extent, thereby leading to variability in gene expression, differentiation, and biological processes [34,36,37]. The 3D-ALi organoids displayed relative low amplitude, but well-synchronized electrical signals, similar to what was observed by extracellular electrode measurements in neural tissues [68]. In contrast, 3D-i organoids showed relatively large amplitude, non-synchronized electrical signals throughout the 50-day study. These signals were significantly more chaotic with a certain resemblance to epileptic EEG patterns [69,70]. This increased electrophysiological variability raises concerns about the safety of using such organoids for cell therapy applications. Moreover, for disease modeling or drug screening purposes, starting with organoids that exhibit such disparity in their base-line electrical activity may introduce confounding variability, potentially limiting the reliability and reproducibility of experimental outcomes. Possible explanations for these non-synchronized currents include i) the size variability (see above), ii) the higher diversity cell types found in immersion organoids, iii) factors such as shear stress or hypoxia, which may increase their excitability [71,72]. Another hypothesis regarding the lack of synchronization in immersion organoids suggests a potential decreased number of GABAergic neurons [73,74]. However, this hypothesis is not supported by sc-RNA seq, which showed a similar proportion of GABAergic neurons in both organoid types (Supplementary Figure S4A). Note, however, that we cannot exclude altered activity or function of these GABAergic.

As highlighted in the introduction, the differentiation of pluripotent stem cells into organoids holds significant potential for a range of applications, including disease modeling, drug screening, and cell therapy. Three-dimensional (3D) cell culture presents several advantages. Firstly, 3D cultures closely mimic the natural in vivo environment, facilitating essential cell-to-cell and cell-to-matrix interactions. This resemblance to physiological conditions reduces the need for frequent cell passaging, which is necessary in 2D cultures and can induce chromosomal alterations. Additionally, 3D cultures simplify protocols and reduce costs—key factors for meeting Good Manufacturing Practice (GMP) standards and addressing broader industrial requirements. Moreover, 3D cultures minimize cell mortality, as they avoid the enzymatic detachment that breaks neurites in 2D cultures, thus protecting neurons within the organoids. However, the commonly used immersion method (3D-i) has significant limitations such as reduced organoid homogeneity due to necessary transfers that create a mix of small and large aggregates. Also, reproducible production of organoids using the immersion method was particularly challenging due to the complex, multistep nature of their generation [29].

The AirLiwell method, described in this manuscript, addresses these limitations by (i) maintaining organoids in microwells without need for transfer; (ii) excellent in vitro long term survival and stability, (iii) preventing organoid to organoid fusion despite the absence of agitation, (iv) assuring oxygen access (air/liquid interface) facilitating neural induction and preventing necrotic centers, (v) ease of medium change and markedly reduced volume of culture media, (vi) high degree of standardization and reproducibility.

## 5. Conclusions

In conclusion, we have developed and validated a new method to produce brain organoids at an air–liquid interface, yielding high numbers of functional neurons. The AirLiwell also offers a high degree of standardization and reproducibility, key requirements for translational and high-throughput applications. These features collectively position the AirLiwell system as a robust and scalable tool platform with promising prospective applications in neurological disease modeling, therapeutic compound screening, and cell-based approaches for disorders such as Parkinson’s disease.

## Figures and Tables

**Scheme 2:**
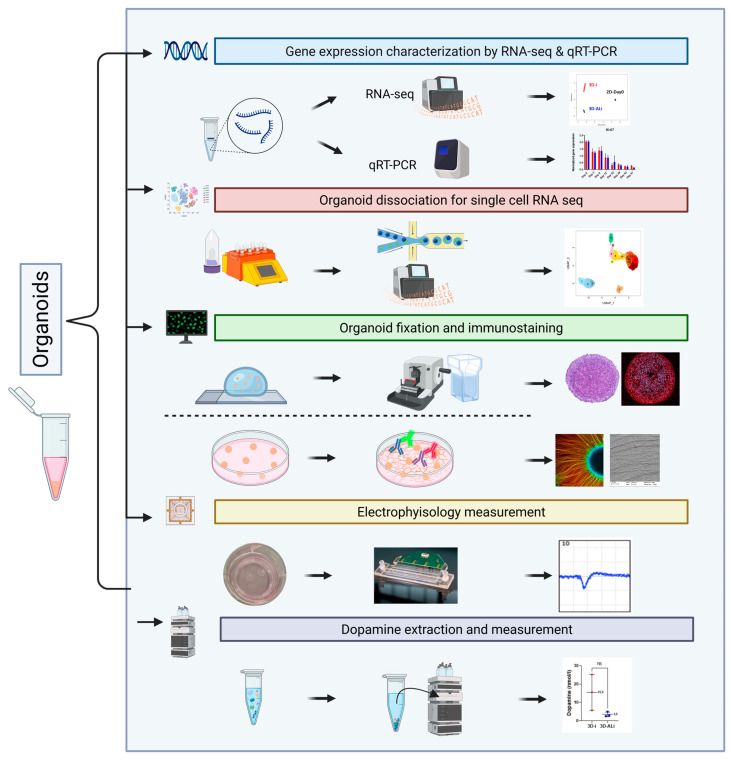
Graphical summary of the second part of material and methods regarding the content analysis of organoids (Created in Biorender. El Harane, S. (2025) https://BioRender.com/16yh18m).

**Table 1:** Primer sequences used for RT-qPCR analysis.

Gene	Primer Sequence (Forward)	Primer Sequence (Reverse)
*Ki-67*	AAGCCCTCCAGCTCCTAGTC	TCCGAAGCACCACTTCTTCT
*MAP-2*	CGAAGCGCCAATGGATTCC	TGAACTATCCTTGCAGACACCT
*TH*	GCACCTTCGCGCAGTTCT	CCCGAACTCCACCGTGAA

## Data Availability

All data generated or analyzed in this study are included in the manuscript and Supplementary Materials.

## Supplementary Materials

The following supporting information can be downloaded at https://www.mdpi.com/article/10.3390/cells14151211/s1. Figure S1: Increase of midbrain specification in 3D-ALi organoids vs. enhancement of neuronal maturation in 3D-i organoids; Figure S2: Gene expression and protein analysis of midbrain organoids in immersion and air-liquid interface cultures; Figure S3: Quantification of immunostaining in plated midbrain organoids and paraffin sections; Figure S4: A second double-blind sc-RNAseq analysis strengthen the first data analysis and interpretation; Figure S5: Single-cell UMAP analysis of midbrain and neuronal marker expression in 3D-i and 3D-ALi organoids; Figure S6: Additional analysis of midbrain organoids cultured using 3D-ALi and 3D-i; Figure S7: Electrical signal in 3D-ALi organoids is highly synchronized; Table S1: 50 most up regulated genes in 3D-i and 3D-Ali at week 6.
