# Supplementary material for: Generation of Individualized, Standardized, and Electrically Synchronized Human Midbrain Organoids
Source: Cells. 2025 Aug 6;14(15):1211. doi: 10.3390/cells14151211 (PMC12346388; doi:10.3390/cells14151211)
Supplement: Supplementary file 1 [file cells-14-01211-s001.zip › supp files/cells-3676337-Supplementaryfigures- revised3.pdf]

Figure S1

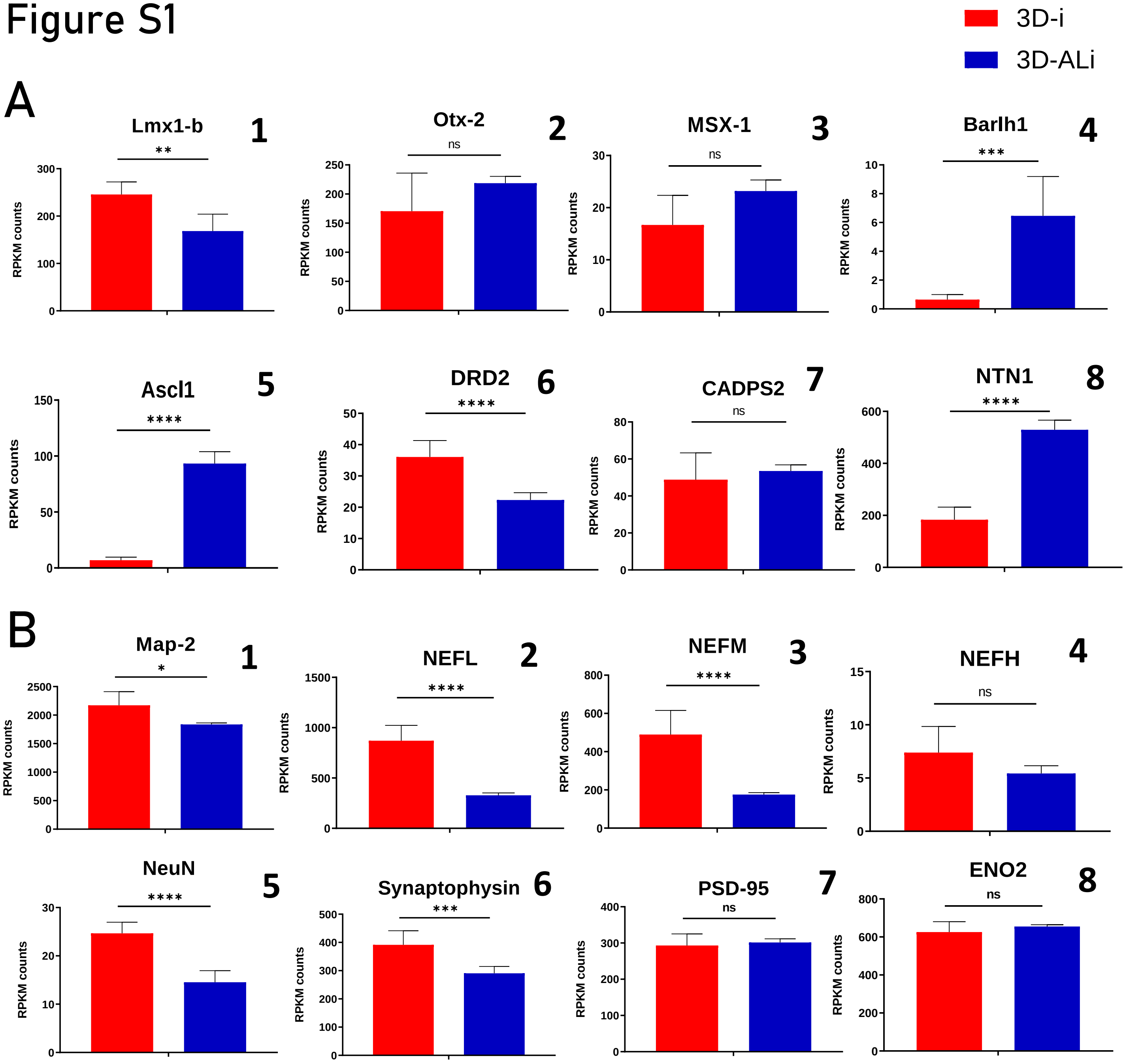

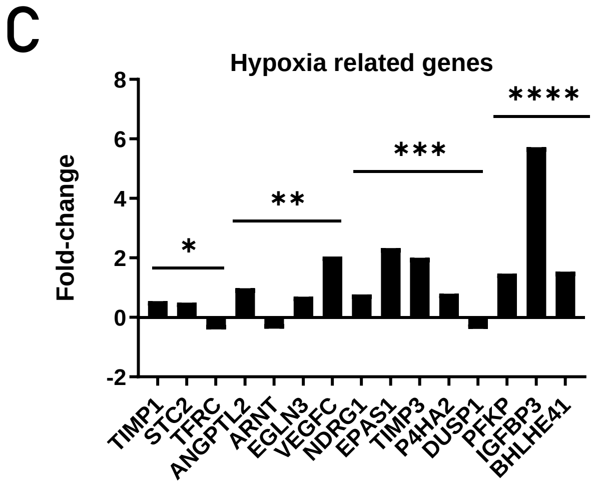

Figure S1: Increase of midbrain specification in 3D-ALi organoids vs enhancement of neuronal maturation in 3D-i organoids

(**A**) Graphs displaying gene expression (in RPKM) of genes involved in dopaminergic neurogenesis and; (**B**) in general neuronal maturation. (**C**) Fold Change Analysis of Hypoxia-Related Genes in ALI vs. Immersion Organoids at Week 6. With statistical significance of all the figure (P-Value = p) is indicated as follows: * $p < 0.05$, ** $p < 0.01$, *** $p < 0.001$, **** $p < 0.0001$ (t-test performed).

Figure S2

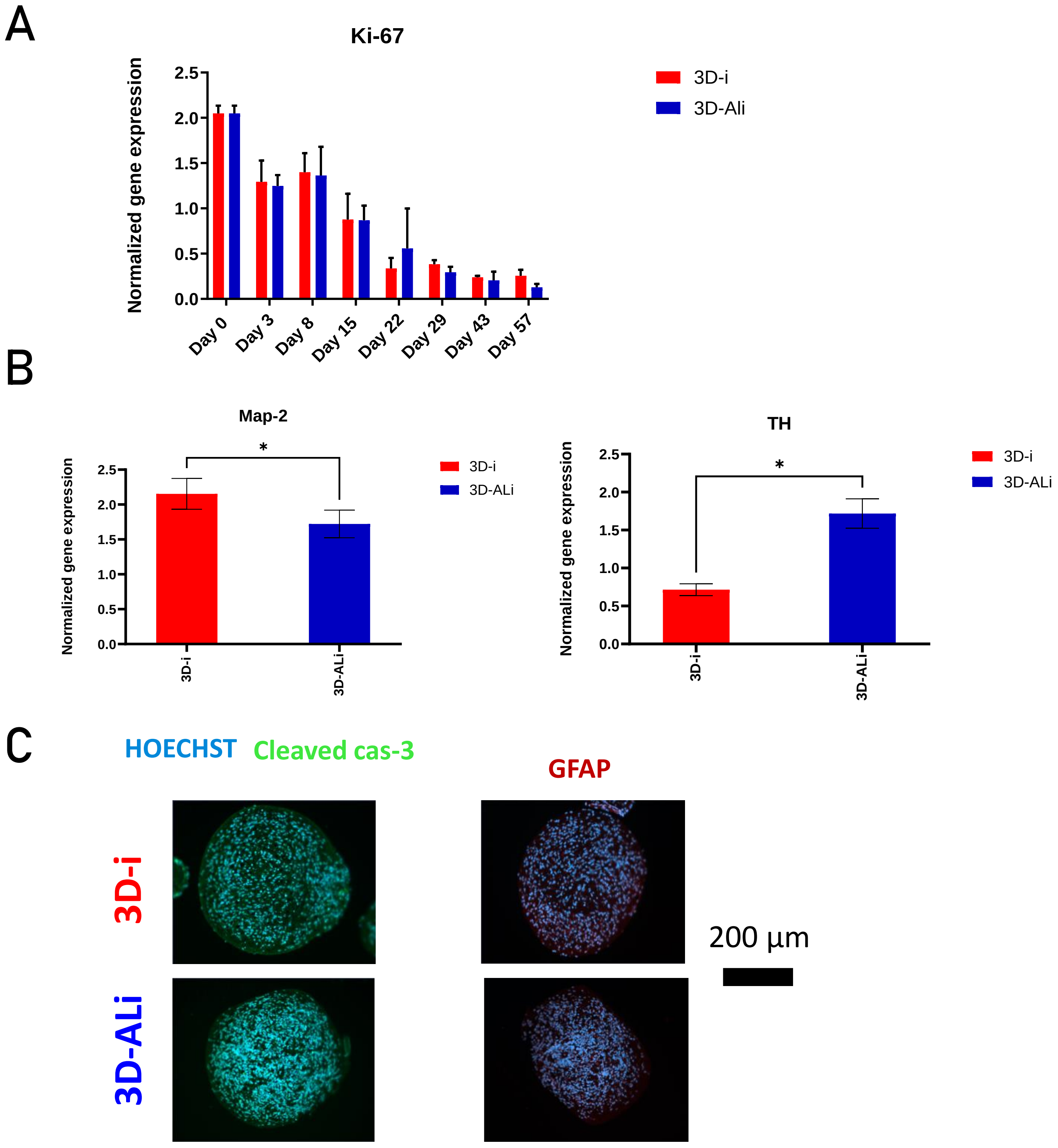

Figure S2 : Gene expression and protein analysis of midbrain organoids in immersion and air-liquid interface cultures

(**A**) qRT-PCR time-course analysis of KI67 gene expression in midbrain organoids cultured in immersion (3D-i) and at the air-liquid interface (3D-ALi) (n=4 independent experiments). (**B**) Gene expression of MAP2 and TH by qRT-PCR at week 6 of differentiation in midbrain organoids cultured in immersion and at the air-liquid interface (n=4 independent experiments). (**C**) Immunostaining showing the expression of cleaved caspase-3 (green), GFAP (red), and Hoechst (blue) in midbrain organoids. The scale bar is indicated in the figure.

Figure S3

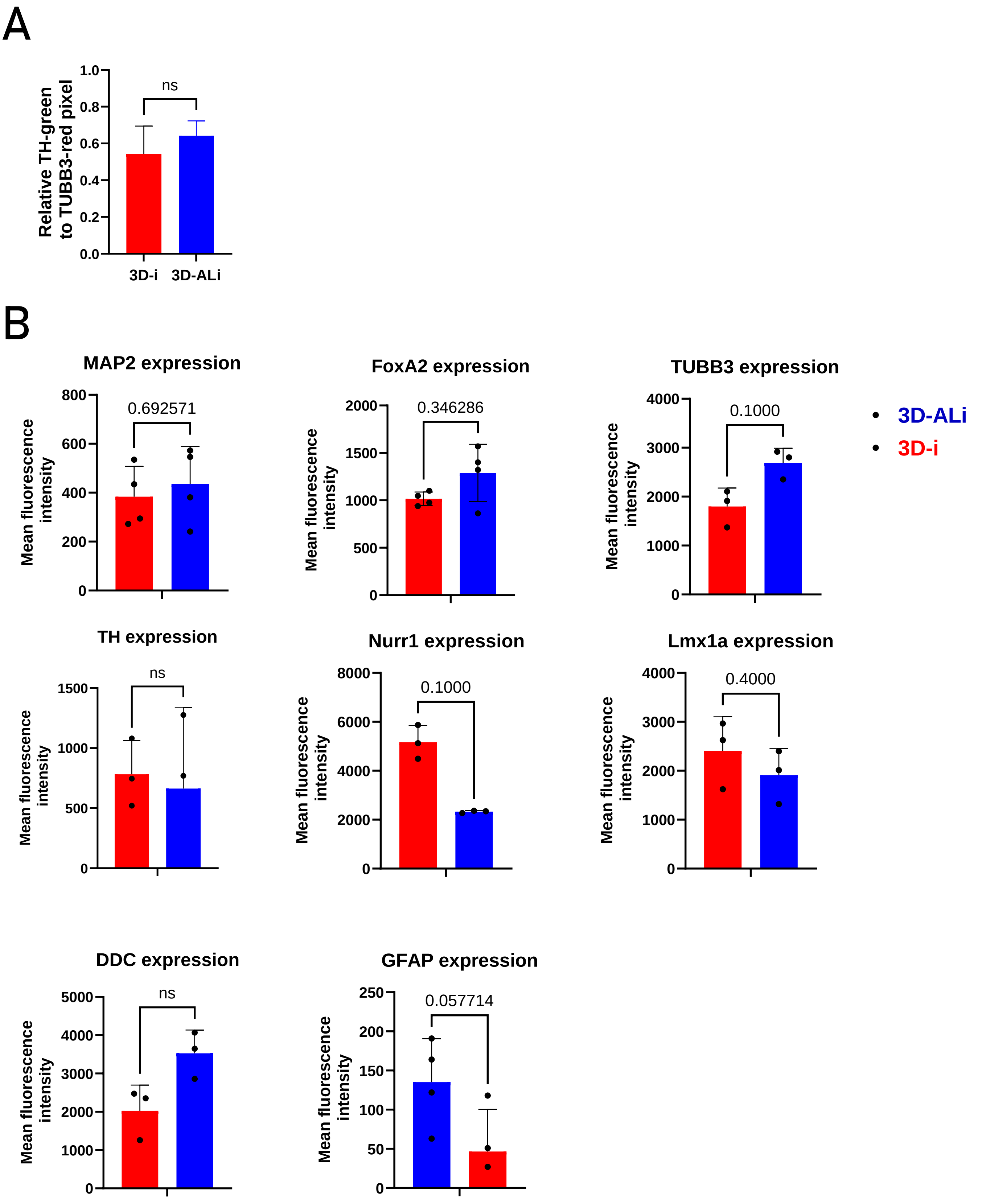

Figure S3 : Quantification of immunostaining in plated midbrain organoids and paraffin sections

(**A**) Quantification of the immunostaining of plated midbrain organoids, showing the relative TH (green) pixel intensity compared to TUBB3 (red) pixel intensity using image J software. (**B**) Quantification of the mean fluorescence intensity of immunostaining on paraffin sections of midbrain organoids, analyzing key markers using image J software.

Figure S4

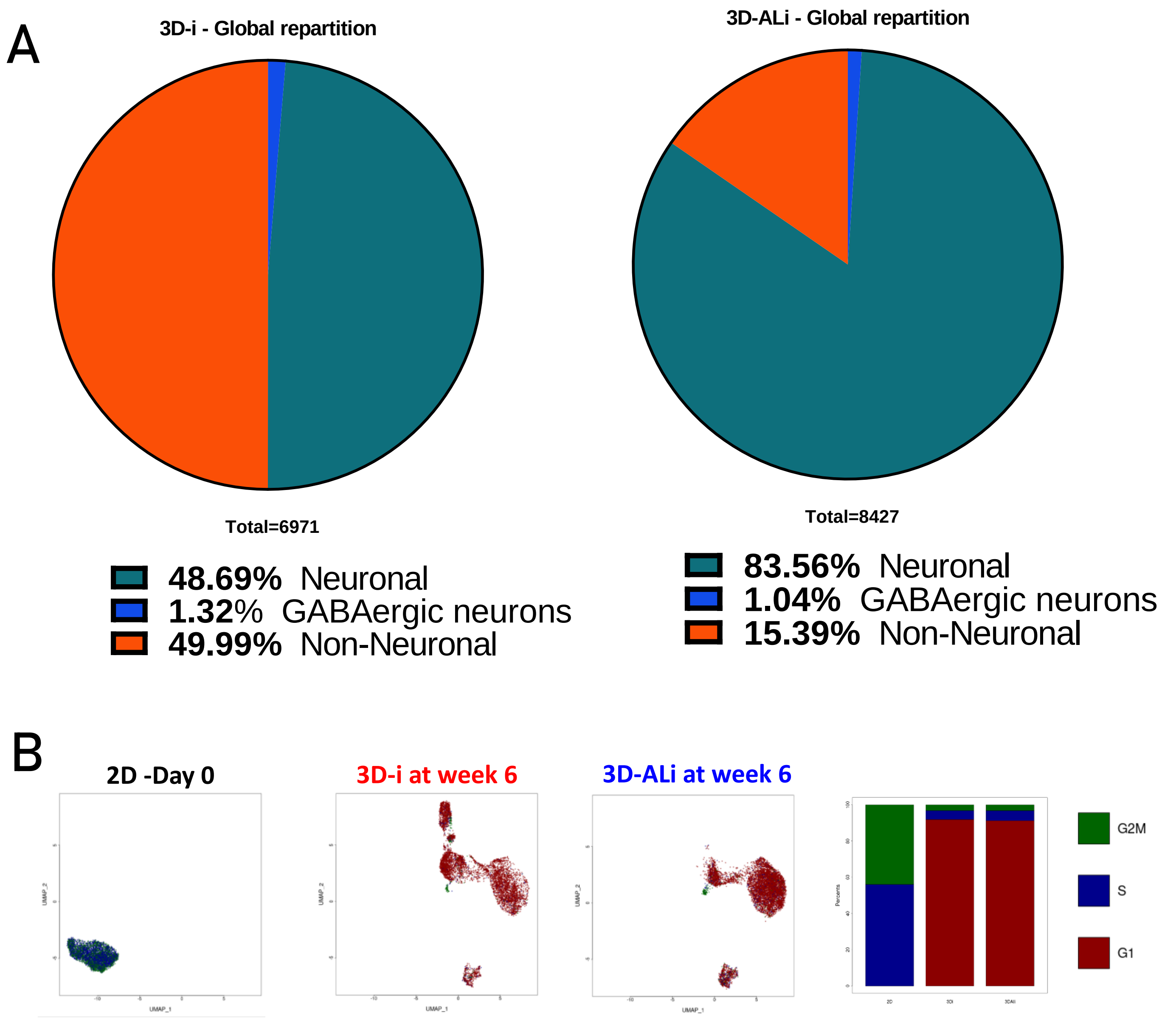

Figure S4: A second double-blind sc-RNAseq analysis strengthen the first data analysis and interpretation

(**A**) Pie charts representing global repartition of cell types found in immersion (left) and in air-liquid interface organoids (right). (**B**) Seurat scoring of cell cycle of cells in 2D cells at Day 0, in 3D-i organoids and in 3D-ALi organoids (from left to right, red color : G1 phase, blue color : S phase, green color : G2/M phase).

Figure S5

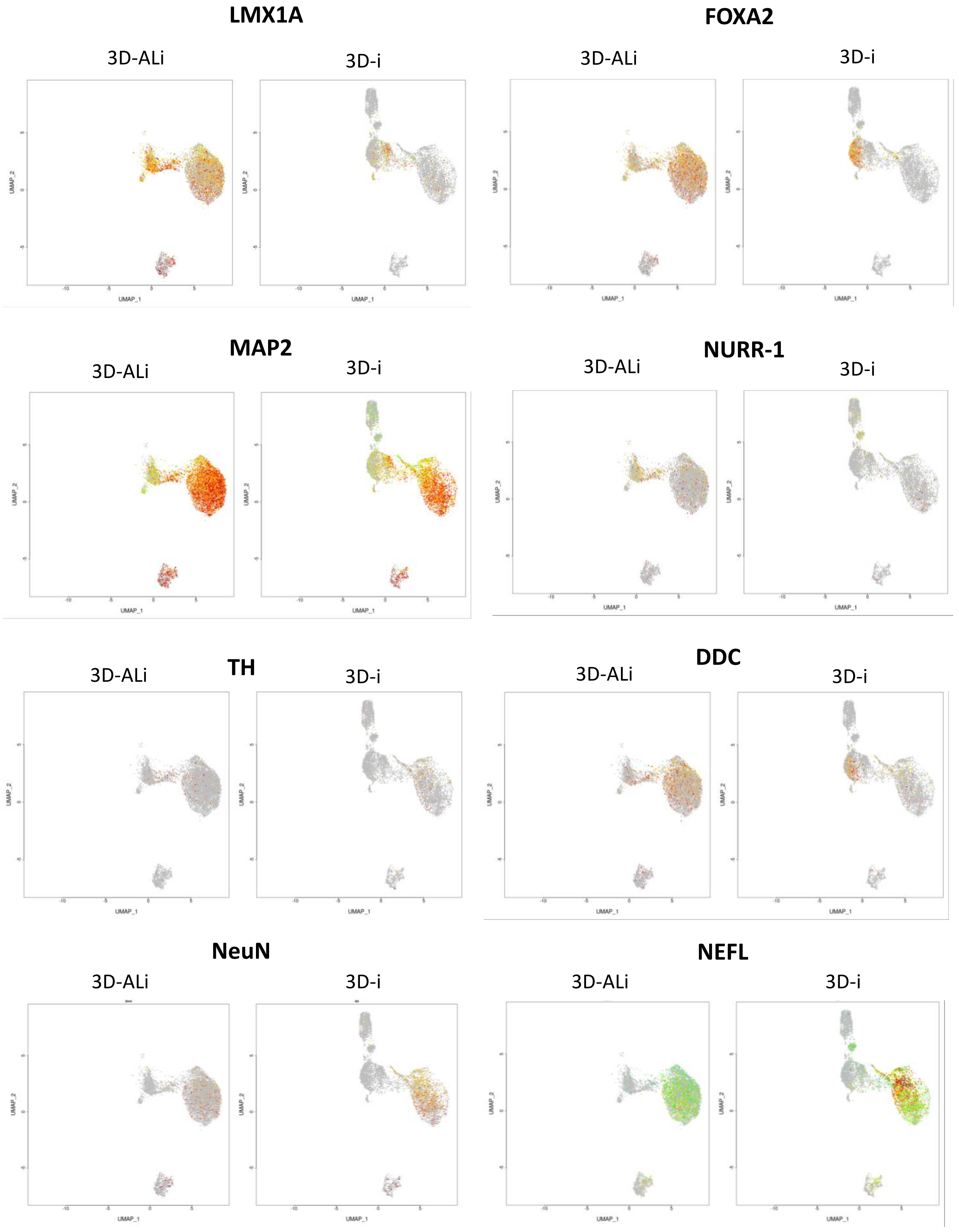
LMX1A
3D-ALi
3D-i
FOXA2
3D-ALi
3D-i
MAP2
3D-ALi
3D-i
NURR-1
3D-ALi
3D-i
TH
3D-ALi
3D-i
DDC
3D-ALi
3D-i
NeuN
3D-ALi
3D-i
NEFL
3D-ALi
3D-i
UMAP_1
UMAP_2

Figure S5 : Single-cell UMAP analysis of midbrain and neuronal marker expression in 3D-i and 3D-ALi organoids

Single-cell expression UMAP (Uniform Manifold Approximation and Projection) analysis showing the expression of key midbrain and neuronal markers in 3D-i and 3D-ALi organoids. The markers analyzed include LMX1A, FOXA2, MAP2, NURR1, TH, DDC, NEUN, and NEFL.

Figure S6

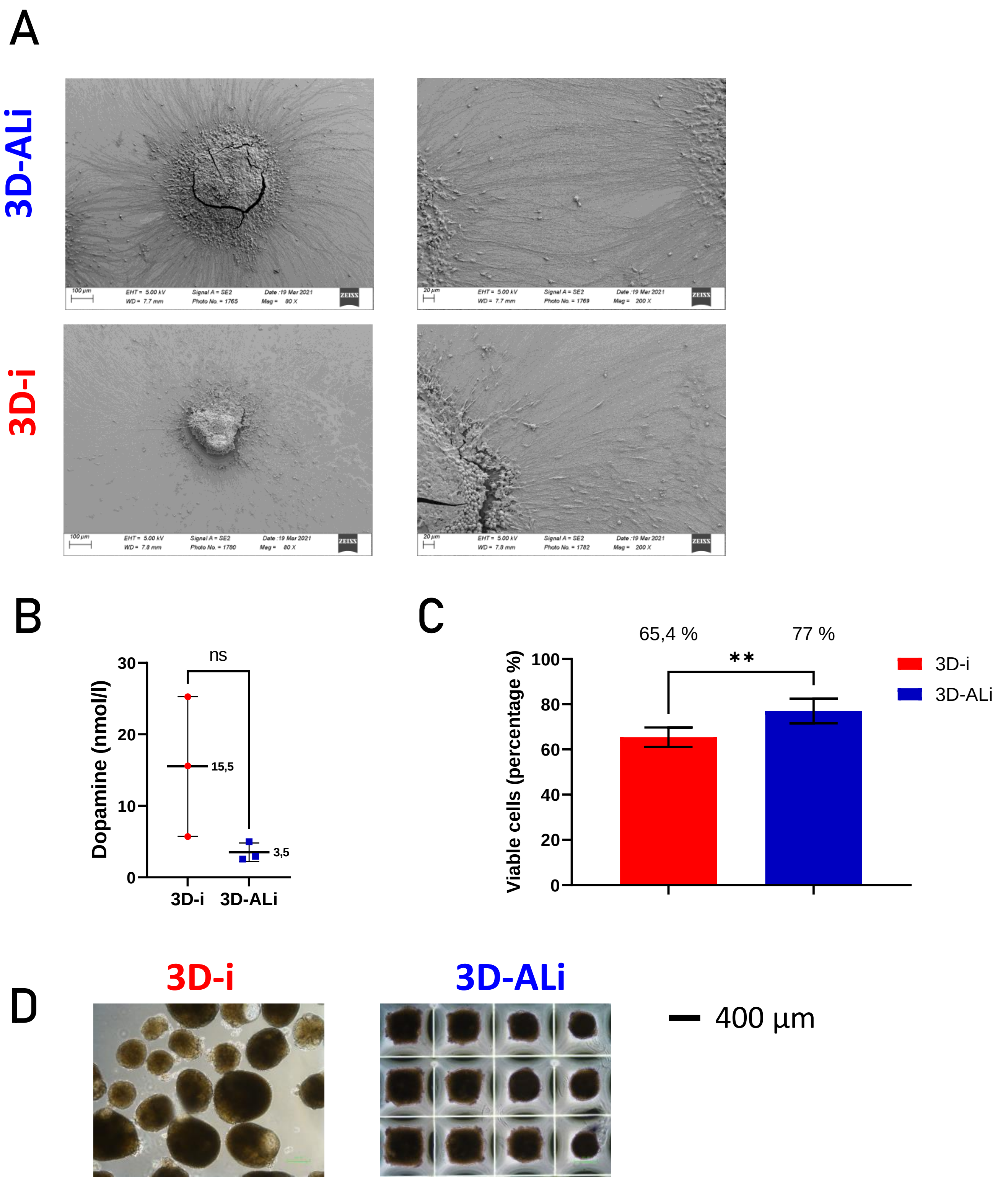

Figure S6: Additional analysis of midbrain organoids cultured using 3D-ALi and 3D-i
(**A**) Supplementary electron microscopy images of midbrain organoids plated on polyornithine/laminin, as shown in Figure 3. (**B**) Quantification of dopamine levels after extraction from 800 midbrain organoids per culture condition, measured by HPLC (n=4 independent experiments). (**C**) Analysis of cell viability percentage in midbrain organoids cultured using 3D-ALi and 3D-i, assessed by FACS (n=3 independent batches). (**D**) Representative images of midbrain organoids at day 135 of culture, maintained using 3D-ALi and 3D-i methods.

Figure S7

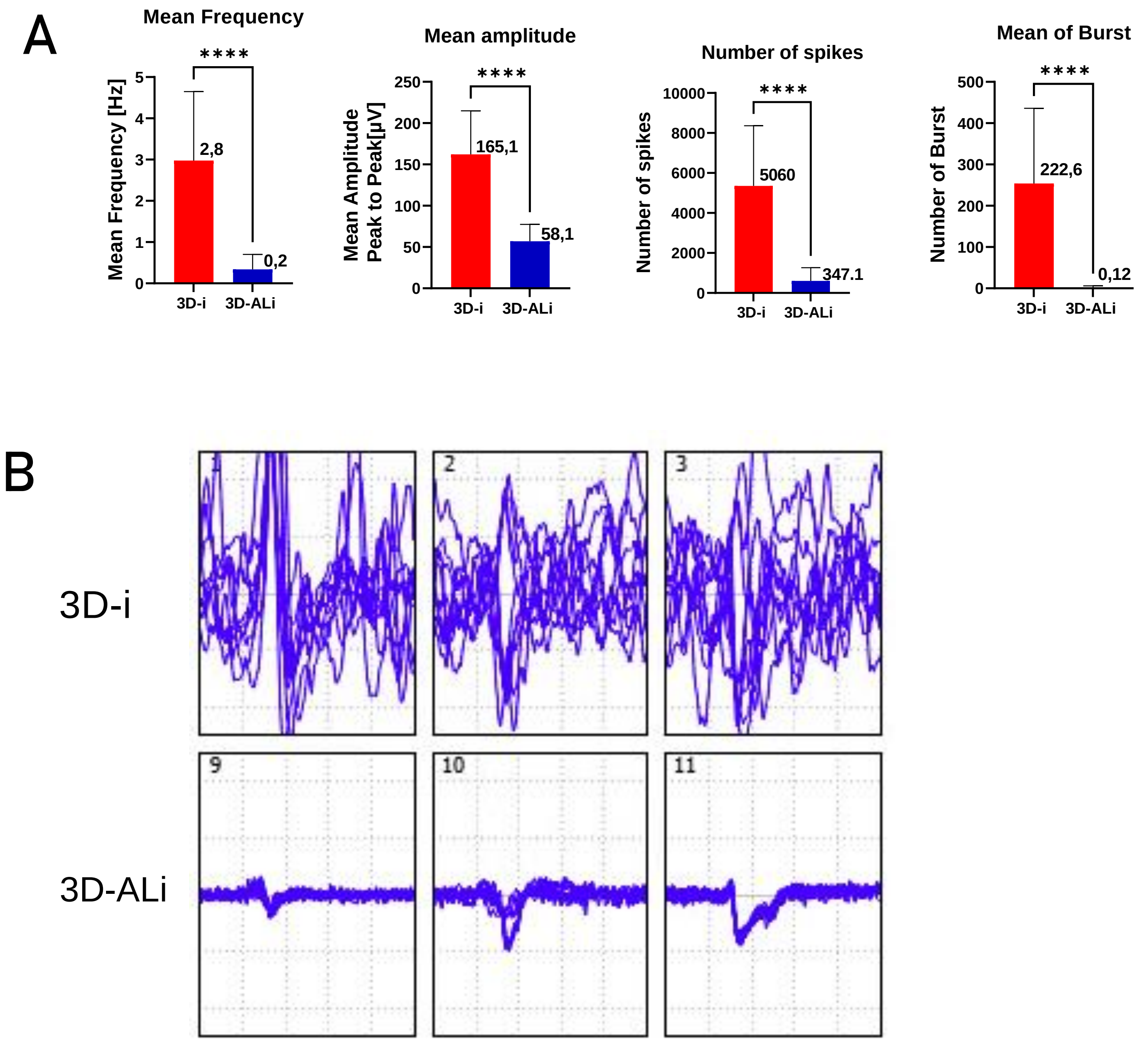

Figure S7: Electrical signal in 3D-ALi organoids is highly synchronized
(**A**) From left to right, graphs showing the mean difference of frequency, amplitude, number of spikes and number of burst overtime between immersion and air-liquid interface organoids ; With statistical significance (P-Value = p) indicated as follows: * $p < 0.05$, ** $p < 0.01$, *** $p < 0.001$, **** $p < 0.0001$ (t-test performed) (**B**) Pictures of the electrical signal overtime and recorded on 8 independent electrodes in immersion organoids (top) and 3D-ALi organoids (bottom).
